# Increased production of BDNF in colonic epithelial cells induced by fecal supernatants from diarrheic IBS patients

**DOI:** 10.1038/srep10121

**Published:** 2015-05-22

**Authors:** Peng Wang, Fei-Xue Chen, Chao Du, Chang-Qing Li, Yan-Bo Yu, Xiu-Li Zuo, Yan-Qing Li

**Affiliations:** 1 Department of Gastroenterology, Qilu Hospital, Shandong University, Jinan 250012, P.R. China; 2 Laboratory of Translational Gastroenterology, Qilu Hospital, Shandong University, Jinan 250012, P.R. China

## Abstract

Colonic brain-derived neurotrophic factor (BDNF) plays an essential role in pathogenesis of abdominal pain in diarrhea-predominant irritable bowel syndrome (IBS-D), but regulation on its expression remains unclear. We investigated the role of fecal supernatants (FSN) from IBS-D patients on regulating BDNF expression in colonic epithelial cells of human and mice. Using human Caco-2 cells, we found that IBS-D FSN significantly increased BDNF mRNA and protein levels compared to control FSN, which were remarkably suppressed by the serine protease inhibitor. To further explore the potential mechanisms, we investigated the impact of protease-activated receptor-2 (PAR-2) on BDNF expression. We found a significant increase in PAR-2 expression in Caco-2 after IBS-D FSN stimulation. Knockdown of PAR-2 significantly inhibited IBS-D FSN-induced upregulation of BDNF. Moreover, we found that phosphorylation of p38 MAPK, not NF-κB p65, contributed to PAR-2-mediated BDNF overexpression. To confirm these results, we intracolonically infused IBS-D or control FSN in mice and found that IBS-D FSN significantly elevated colonic BDNF and visceral hypersensitivity in mice, which were both suppressed by the inhibitor of serine protease or antagonist of PAR-2. Together, our data indicate that activation of PAR-2 signaling by IBS-D FSN promotes expression of colonic BDNF, thereby contributing to IBS-like visceral hypersensitivity.

Irritable bowel syndrome (IBS) is a common chronic functional disorder of the gastrointestinal tract. Abdominal pain, the most debilitating aspect to IBS patients, leads to a poor quality of life^1^. Visceral hypersensitivity has been revealed to be one of the major mechanisms of abdominal pain in IBS^2^. Lines of studies have demonstrated that the increased mucosal mediators, such as serotonin and histamine that are released by activated intestinal immune cells, are important contributors to the development of visceral hypersensitivity^3^,^4^. In a previous study, we have discovered another pain mediator, brain-derived neurotrophic factor (BDNF), which has been well-recognized as an essential modulator in central physiologic and pathologic pain^5^, is substantially increased in colonic epithelium and lamina propria in patients with IBS, especially in diarrhoea-predominant IBS (IBS-D) subgroup. The over-expressed colonic BDNF is significantly correlated with abdominal pain symptoms of IBS-D^6^. Notably, enteric BDNF has been revealed to enhance responses of enteric neurons to pain-related neurotransmitters such as substance P and serotonin^7^. Thus, these findings indicate an important role of enteric BDNF in facilitation of pain in IBS. However, how the expression of BDNF is regulated in intestinal epithelial cells (IEC) remains unclear.

Convincing data have revealed that fecal serine protease activity is markedly elevated in patients with IBS-D, which is responsible for the increased intestinal permeability and subsequent visceral hypersensitivity through a mechanism of protease activated receptor-2 (PAR-2) activation^8^,^9^,^10^,^11^. Of note, PAR-2 action has also been shown to be involved in secretory process of epithelial cells^12^,^13^,^14^. Along this line, whether the altered expression of intestinal epithelial BDNF in IBS-D patients can be attributed to the elevated fecal serine protease activity and subsequent activation of PAR-2 is worth further investigation.

Therefore, we conducted this study to examine the effect of colonic luminal fecal supernatants from IBS-D patients on expression of colonic epithelial BDNF, and the potential mechanism of how its release is regulated.

## Results

### Fecal serine protease activity of IBS-D fecal supernatants (FSN)

**Figure 1:**
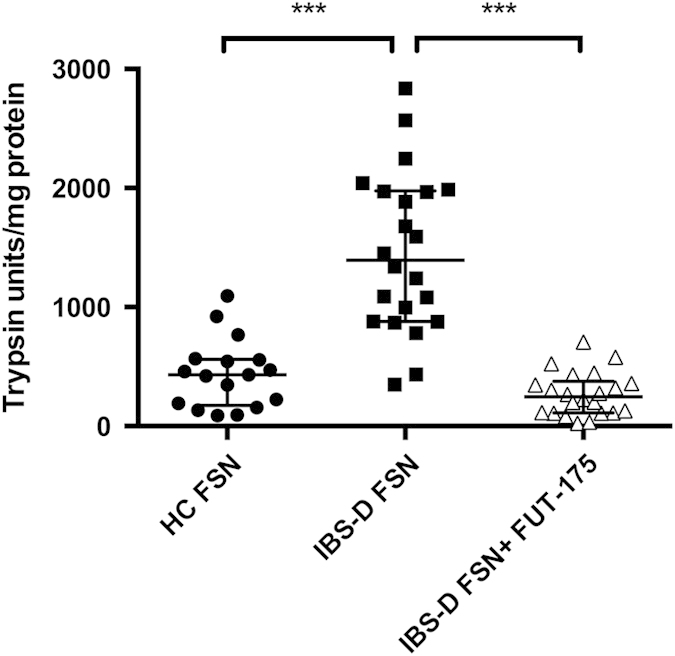
Serine protease activity was increased in fecal supernatants (FSN) from IBS-D patients. The serine protease inhibitor FUT-175 markedly inhibited protease activity released from IBS-D FSN (n = 17 for HC FSN, n = 22 for IBS-D FSN. ^***^P < 0.001).

Total fecal protease activity in IBS-D patients (1461 ± 143.1 U/mg of protein) was approximately 3-fold greater than that in healthy controls (HCs) (438.6 ± 70.2 U/mg of protein). Preincubation of IBS-D FSN with the serine protease inhibitor FUT-175 significantly reduced the proteolytic activity close to that of HCs, which suggests that the increased proteolytic activity in IBS-D FSN is dependent on serine protease (Fig. 1).

### IBS-D FSN triggered upregulation of BDNF mRNA and protein levels in human colonic epithelial cell line Caco-2

**Figure 2:**
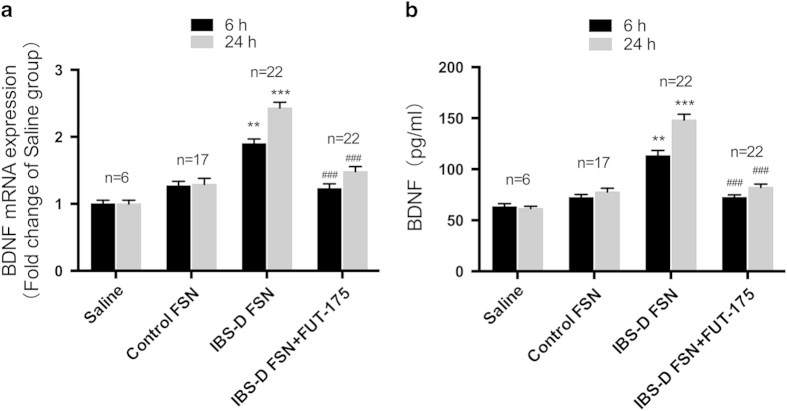
IBS-D fecal supernatants (FSN) elevated expression of BDNF in Caco-2 cells. (**a**) BDNF mRNA levels. (**b**) BDNF protein levels. Preincubation of IBS-D FSN with FUT-175 significantly attenuated the effect of IBS-D FSN on BDNF expression in Caco-2 cells. (^**^P < 0.01, ^***^P < 0.001 vs. saline; ^##^*P* < 0.01, ^###^*P* < 0.001 vs. saline).

BDNF mRNA expression was increased by 1.4- fold at 6 h and 1.8-fold at 24 h following IBS-D FSN treatment, compared to control FSN (Fig. 2a). Preincubation of IBS-D FSN with FUT-175 abolished the effect of IBS-D FSN on BDNF mRNA expression. Similarly, ELISA showed that BDNF protein levels were 1.7-fold higher at 6 h and 2-fold higher at 24 h in culture supernatants from IBS-D FSN-treated cells than control FSN, with these effects prevented by FUT-175 either (Fig. 2b).

### PAR-2 knockdown suppressed IBS-D FSN-induced BDNF overexpression in Caco-2 cells

**Figure 3:**
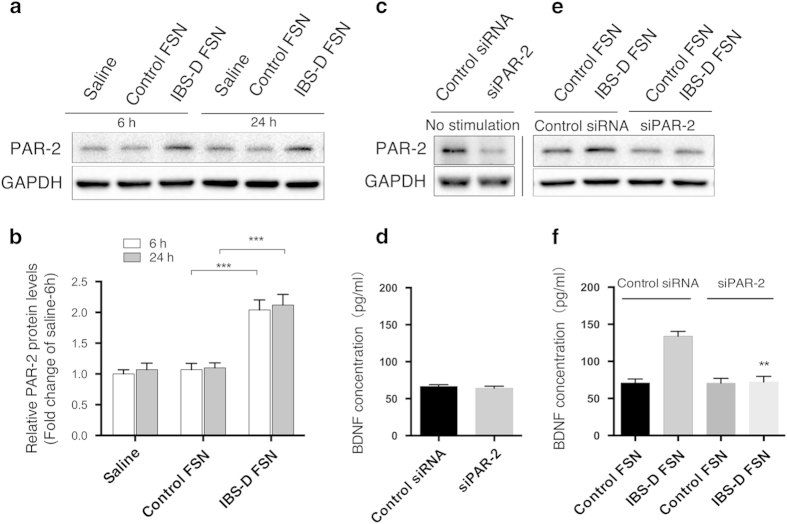
Protease-activated receptor-2 (PAR-2) was involved in IBS-D fecal supernatants (FSN)-induced BDNF overexpression in Caco-2 cells (**a** and **b**) Western blotting of PAR-2 in Caco-2 cells (*** *P* < 0.001). (**c**) siRNA-mediated PAR-2 knockdown in Caco-2 cells. (**d**) BDNF protein levels secreted by Caco-2 cells after PAR-2 knockdown. (**e**) Effect of IBS-D FSN on PAR-2 expression in siRNA-transfected Caco-2 cells. (**f**) BDNF protein levels secreted by Caco-2 cells after PAR-2 knockdown followed by IBS-D FSN stimulation (^**^ *P* < 0.01, PAR-2 siRNA- vs. control siRNA-transfected cells after IBS-D FSN treatment). The gels were run under the same experimental conditions. Cropped gels/blots are presented (full-length gels/blots are shown in suppl. Fig. S1 with indicated cropping lines).

IBS-D FSN induced a markedly increase in PAR-2 protein levels of Caco-2 cells at 6 h and 24 h (Fig. 3a,b). To further validate whether PAR-2 activation is involved in IBS-D FSN-induced BDNF upregulation, we performed lipofectamine-mediated RNA interference to silence PAR-2 in Caco-2 cells. Results showed that siRNA against PAR-2 reduced PAR-2 protein levels by nearly 75% (Fig. 3c) but did not alter the basal expression of BDNF in Caco-2 cells (Fig. 3d). However, PAR-2 knockdown significantly repressed IBS-D FSN-induced upregulation of BDNF compared to control FSN (Fig. 3e,f).

### p38 MAPK but not p65 NF-κB was associated with IBS-D FSN-mediated BDNF upregulation

**Figure 4:**
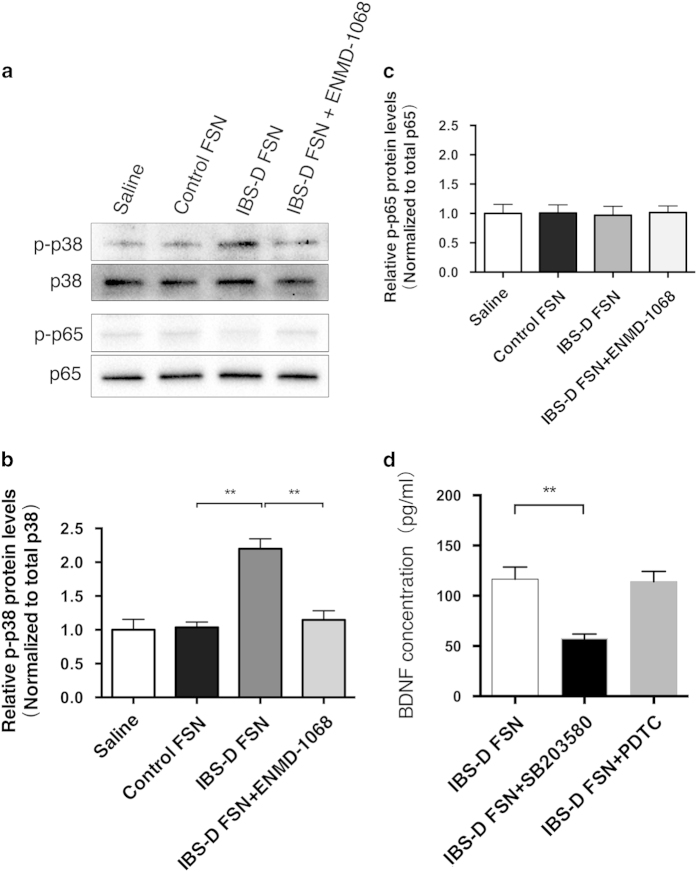
Phosphorylation of p38 MAPK but not p65 NF-κB signaling contributed to IBS-D fecal supernatants (FSN)-triggered BDNF expression in Caco-2 cells. (**a b** and **c**) Western blotting of phospho-p38, phospho-p65, total p38 and total p65 protein levels in Caco-2 cells. (**d**) ELISA analysis of BDNF levels in Caco-2 cells after treatment with IBS-D FSN, and preadministration of SB203580 or PDTC followed by IBS-D FSN stimulation (^**^ *P* < 0.01). The gels were run under the same experimental conditions. Cropped gels/blots are presented (full-length gels/blots are shown in suppl. Fig. S1 with indicated cropping lines).

According to the available literature, PAR-2 activation in Caco-2 cells activates p38 MAPK to facilitate IL-8 expression without directly activating NF-κB^12^. Thus, to further identify the downstream pathway of PAR-2 activation in inducing BDNF overexpression in Caco-2 cells, we focused on MAPK p38 and NF-κB p65. We found that IBS-D FSN induced a rapid phosphorylation of p38 MAPK after 15 min of stimulation, although total p38 MAPK appeared unchanged. Preadministration of Caco-2 with PAR-2 selective inhibitor ENMD-1068 clearly blocked the effect of IBS-D FSN on p38 phosphorylation (Fig. 4a,b). However, we did not observe a significant change in levels of either phosphorylated p65 or total p65 before and after administration of IBS-D FSN (Fig. 4c). Furthermore, pretreatment with specific inhibitor of p38-MAPK SB203580, but not NK-κB specific blocking agent PDTC, prevented IBS-D FSN elevating BDNF protein levels (Fig. 4d).

Intracolonic infusion of IBS-D FSN induced overexpression of colonic BDNF and visceral hypersensitivity in mice, which were suppressed by inhibitors of serine protease, PAR-2 or BDNF

**Figure 5:**
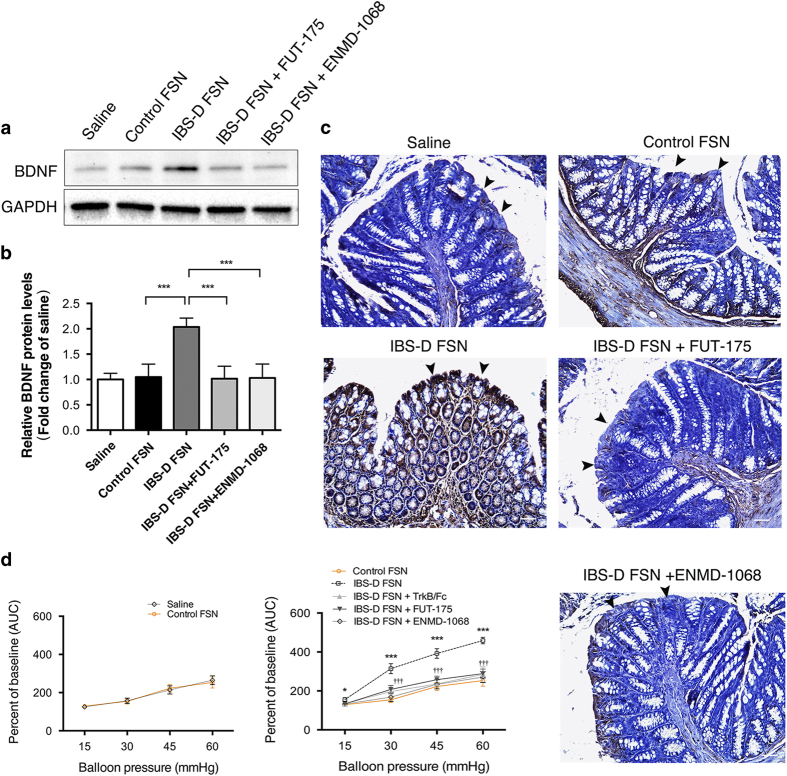
IBS-D fecal supernatants (FSN) induced colonic BDNF overexpression and visceral hypersensitivity in mice. (**a** and **b**) Western blotting of BDNF expression in colon of mice (***P < 0.001). (**c**) Immunohistochemical staining of BDNF in epithelium (Black arrowheads) and mucosa in colon of mice (Bars: 50 μm). (**d**) Measurement of visceral sensitivity to colorectal distension in mice by using electromyography recording (^*^P < 0.05, ^***^P < 0.001 IBS-D FSN vs. control FSN; ^†††^*P* < 0.001, IBS-D FSN + FUT-175 or ENMD-1068 or TrkB/Fc vs. IBS-D FSN; n = 8 per group; AUC: area under curve). The gels were run under the same experimental conditions. Cropped gels/blots are presented (full-length gels/blots are shown in suppl. Fig. S1 with indicated cropping lines).

Next we aimed to further confirm the effect of IBS-D FSN on colonic BDNF expression *in vivo*. We stimulated colonic epithelium of mice by intracolonic administration of selected IBS-D FSN (a serine-protease activity greater than 1093 U/mg protein (the largest activity in HC group)), control FSN or saline for 1 h. Control FSN had no specific effect on BDNF expression as compared with saline (Fig. 5a,b). BDNF levels in colon of mice nearly doubled after stimulation by IBS-D FSN compared to control FSN. IBS-D FSN failed to upregulate BDNF expression in colon of mice when preincubated with FUT-175. In mice pretreated with ENMD-1068, IBS-D FSN could not induce elevation of colonic BDNF either. Immunochemistry staining confirmed the western blotting results and showed that the increased BDNF was mainly observed in colonic epithelium and mucosa (Fig. 5c).

To further gain a functional profile of IBS-D FSN in mouse colorectal sensitivity, we performed electromyography recording (EMG) to record the abdominal muscle response to colorectal distension (CRD) after intracolonic infusion of IBS-D FSN, control FSN or saline. As shown in Fig. 5d, control FSN did not alter colorectal sensitivity to CRD compared to saline. Strikingly, IBS-D FSN significantly increased the EMG response to CRD in comparison to control FSN at CRD pressures of 15, 30, 45 and 60 mmHg. In contrast, IBS-D FSN-induced increases in EMG records were significantly repressed in mice pretreated with either the endogenous BDNF sequester TrkB/Fc, FUT-175 (preincubation with IBS-D FSN), or ENMD-1068.

## Discussion

Overwhelming evidences have shown that BDNF plays a crucial role in regulating synaptic plasticity in brain and spinal cord, which participates in central sensitization in various types of pain process^15^,^16^,^17^. Although effects of BDNF in gut are only beginning to be discovered, there has been growing evidence that BDNF also plays an important role in gastrointestinal function. BDNF could augment neuronal responsiveness to excitatory neurotransmitters such as 5-HT (5-hydroxytryptamine), substance P and CGRP (Calcitonin gene-related peptide), leading to a facilitation of release of synaptic vesicles from the enteric nervous system^7^,^18^. The promoting effect of BDNF on enteric synaptic efficacy might help explain how the increased BDNF in colon of IBS-D patients contributes to abdominal pain. However, the cause of BDNF overexpression in colon is still completely unknown. Immunohistochemistry staining showed that the increased BDNF in colon mainly localized in epithelial cells and lamina propria, leading us to speculate that this event likely originates from colonic epithelium^6^. In the present study, we showed that Caco-2, a human colonic cancer cell line, was responded significantly to stimulation of IBS-D FSN, followed by a significantly increased BDNF release. This indicates an involvement of luminal contents in initiating BDNF release from IEC in IBS-D patients. Recent studies by others found that IBS-D patients have a significantly increased serine protease activity in supernatants from luminal contents, compared with healthy subjects, constipation-predominant IBS (IBS-C) or alternating type IBS (IBS-A) patients^8^,^9^,^10^,^11^. The elevated serine protease activity can activate PAR-2 that is expressed abundantly at the apical side of IEC, which changes the functional status of IEC such as inducing phosphorylation of myosin light chain (MLC), degrading tight junction proteins and finally increasing intestinal permeability^10^,^19^,^20^. This concept has been proposed to be one crucial mechanism of visceral hypersensitivity in IBS-D. While as mentioned above, PAR-2 receptor has also been shown to drive many secretory processes, thus, there is possibility that PAR-2 activation is responsible for the altered secretion of BDNF from IEC when stimulated by IBS-D FSN. As expected, we observed that the increased release of BDNF from Caco-2 could be inhibited by seine protease inhibitor. Moreover, knockdown of PAR-2 by siRNA lead to a failure of IBS-D FSN in elevating BDNF expression in Caco-2 cells. These data confirmed what we had thought that BDNF release is dependent on PAR-2 activation. Although our findings of PAR-2-mediated BDNF overexpression in epithelial cells has never been tested by others, they are supported by recent data in dorsal horn showing that PAR2-activation can upregulate BDNF, which contributes to central sensitization in rats with bone cancer pain^21^.

Colon cancer cell line Caco-2 showed a positive response to stimulation of IBS-D FSN, while whether normal intestinal epithelial cells *in vivo* have a similar response needs further verification. Therefore, we designed experiments on mice to examine the relationship between IBS-D FSN stimulus, colonic BDNF expression, PAR-2 activation and visceral sensitivity. Our study has confirmed previous reports that IBS-D FSN usually comes with a higher serine protease activity than control FSN, which can induce colonic hypersensitivity in mice rapidly^10^. As expected, intracolonic infusion with IBS-D FSN significantly increased BDNF expression in mouse colon, especially in epithelial cells and lamina propria, compared to control FSN or saline. This effect was diminished by either serine protease inhibitor or PAR-2 antagonist, which further supports the possibility that alteration of colonic BDNF is due to the increased fecal serine protease activity and PAR-2 activation. Previous results showed that exogenous BDNF exerted a dose-dependent effect on decreasing the threshold pressure during CRD in mice^6^. In colonic mucosa, the BDNF high affinity receptor TrkB has been demonstrated to be expressed in enteroglial cells and intestinal mucosal nerve terminals^7^,^22^. These mucosal nerve terminals, which are projected either from thoracolumbar pathways that have cell bodies in thoracolumbar dorsal root ganglia (DRG) or lumbosacral pathways that have cell bodies in lumbosacral DRG, are sensitive to mediators released from various types of of mucosal enteroendocrine cells^23^. We and others have previously recognized these increased mucosal nerve terminals in colonic mucosal biopsies of IBS patients and, in the colonic mucosa where these nerve terminals can be seen, increased BDNF immunostaining can be observed as well, suggesting that mucosal BDNF is in close proximity to these sensory nerve terminals^4^,^6^. Upon activation, TrkB-mediated signaling could facilitate release of 5-HT and CGRP and amplify neuronal response to excitatory neurotransmitters such as 5-HT and substance P, thereby promoting sensitization of sensory nerve fibers^7^,^18^. In the present study, we showed that blocking BDNF signaling in colon by intracolonic preadminstration of TrkB/Fc largely attenuated the IBS-D FSN-induced enhancement of abdominal muscular response to CRD in mice, which confirmed the notion that increased BDNF in gut contributes to IBS-like visceral hypersensitivity^6^. In addition, serine protease inhibitor and PAR-2 antagonist could repress the effect of IBS-D FSN on visceral sensitivity, further supporting the concept that PAR-2 activation is involved in visceral hypersensitivity.

The downstream pathway of PAR-2 in modulating cell secretion is complex and shows a big heredity among different cell types. For example, in spinal cord, the upregulation of BDNF induced by PAR-2 activation involves NF-κB p65 activation.^21^ However, in Caco-2 cells, PAR-2 activation was demonstrated to correlate with phosphorylation of p38 MAPK but not p65 NF-κB^12^. To make a clear understanding of the signaling pathway that modulates BDNF expression in Caco-2 cells, we tested both p38 MAPK and p65 NF-κB activity. Of note, an enhanced phosphorylation of p38 but not p65 was induced by IBS-D FSN, which could be prevented by PAR-2 antagonist ENMD-1068. Meanwhile, the increased BDNF levels could also be significantly downregulated by p38 specific inhibitor SB203580 but not NF-κB inhibitor PDTC. Thus, these results are in agreement with the previous reports about the close association between PAR-2 and p38 MAPK activation. Also, our data are in line with the findings in central system that p38 MAPK is required for production of BDNF in rat hippocampus or microglia^24^,^25^. Collectively, we can speculate that p38 MAPK phosphorylation likely contributes to IBS-D FSN-induced BDNF expression in Caco-2 epithelial cells.

A main limitation of this study is that the PAR-2 antagonist ENMD-1068 is not potential enough, leading to its limitation for laboratory and clinical use. Thus, further studies using GB88 (a more potent and specific PAR-2 antagonist)^26^,^27^ and intestinal epithelium-specific PAR-2 knockout mouse model should be carried out to clarify the role of epithelial PAR-2 on visceral hypersensitivity.

In summary, we propose that release of BDNF in colonic epithelial cells can be regulated by PAR-2-p38 MAPK signaling and the increased fecal serine protease activity in IBS-D patients can initiate this event. These results suggest that apart from altering intestinal permeability, IBS-D FSN-mediated PAR-2 activation can also contribute to IBS-like visceral hypersensitivity through regulating release of BDNF from intestinal epithelium.

## Methods

### Fecal supernatants (FSN) collection

All experimental protocols for the human study were approved by the Clinical Ethical Committee of Qilu Hospital of Shandong University (Document No.12113). All subjects gave their written informed consent prior to enrollment, and all methods used in the human study were carried out in accordance with the approved guidelines.

We invited healthy subjects and IBS-D patients to participate in this study in the Department of Gastroenterology of Qilu Hospital. All IBS-D patients were diagnosed and subclassified according to the ROME III criteria on the basis of the predominant stool pattern^28^. In addition, each subject received a work-up to exclude organic diseases including other causes of diarrhea. This work-up included medical history taking, physical examinations, detailed blood and stool analysis (e.g., excluding infectious diarrhea), serological assays for celiac disease (detection of antitransglutaminase and antiendomysial antibodies) and colonoscopy with a mucosal biopsy for histology (e.g., excluding lymphocytic colitis). Subjects were also excluded if they were taking any drugs that might influence this study, or had an abdominal surgery history or any other organic gastrointestinal disorders. HCs were individuals who received negative results after stool and blood tests and colonoscopy for colorectal cancer screening.

Fecal samples were obtained from each subject and transported to Qilu hospital within 1 hour. Each sample was dissolved and homogenized in 0.9% NaCl solution (1g/7 ml for animal study and 1 g/3.5 ml for cell culture) on ice. Supernatants were collected after centrifugation (4500 rpm, 4 °C, 10 min), and filtered by 0.8 μm-sized filters and stored at −20 °C. Serine-protease activity was detected based on Gecse’s method^10^. We incubated FSN (25 μl) with 1 ml of reaction buffer (20 mM Tris-HCl and 0.15 M NaCl; PH 8.3) and 1 ml of 0.5% (w/v) azo-casein at 40 °C. Trichloracetic acid (1 ml; 10% (v/v)) was used to stop the reaction after 20 min, followed by centrifugation. Supernatants were collected for absorbance measurement at 366 nm. To verify whether the fecal protease is serine protease-specific, IBS-D FSN were preincubated with serine protease inhibitor FUT-175 (50 ug/ml) for 30 min before protease activity measurements. Protease activity was normalized against protein concentration and quantitated against bovine trypsin calibrate (expressed as trypsin units/mg protein).

### Caco-2 cells and reagents

Caco-2, a human colorectal carcinoma-derived epithelial cell line (ATCC, Manassas, VA, USA), were cultured in high glucose DMEM supplemented with 10% FBS (Gibco CA, USA) in a humidified incubator (5% CO2, 37 °C). Cells were seeded in Transwell^®^ inserts (pore size: 0.4 μm, diameter: 12 mm, Costar), serum-starved for 24 h, and then stimulated with saline, control FSN, IBS-D FSN and IBS-D FSN preincubated with FUT-175 (30 min, 50 μg/ml) for 6 h and 24 h respectively. To determine the involvement of PAR-2, p38 mitogen-activated protein kinase (MAPK) and p65 nuclear factor kappa B (NF-κB) in the action of IBS-D FSN, cells were pretreated with PAR-2 antagonist (ENMD-1068, 5 mM, Sigma), p38 MAPK inhibitor (SB203580, 1 μM, Sigma) and NF-κB inhibitor (PDTC, 100 μM, Sigma) for 30 min followed by a replacement with fresh medium containing IBS-D FSN, respectively.

### PAR-2 gene silencing

Small interfering RNA (#1960, Ambion) against human PAR-2 and a control siRNA (AM4611, Ambion) were used to transfect Caco-2 cells. Lipofectamine RNAiMAX (Invitrogen) was used to perform reverse transfection following the manufacturer’s protocols. Cells at 24 h after transfection were stimulated by IBS-D or control FSN for another 24 h.

### Real-time quantitative PCR (qPCR)

Total RNA was extracted by using Trizol (Invitrogen, San Diego, CA, USA). Total RNA (1 μg) was reverse transcribed into cDNA by using ReverTra Ace® qPCR RT Kit (TOYOBO, Japan) in a Mastercycler thermal cycler (Eppendorf, German). Real time qPCR was performed using SYBR Green reagent (Takara, Japan) in a fluorescence thermocycler (LightCycler; Roche Diagnostics, Mannheim, Germany). Primers for human BDNF gene were as follows: forward 5′-AGGTGGCTCTGGAATGACAT-3′ and reverse 5′-GGCATAAGTCGGCTTGAGTG-3′. BDNF mRNA levels were normalized to those of human GAPDH.

### Enzyme-linked immunosorbent assay (ELISA)

Culture supernatants were collected 6 h and 24 h after treatment with IBS-D or control FSN. BDNF protein levels were measured by a commercially available ELISA kit (Promega, Madison, WI, USA).

### Western blotting

Total proteins were extracted and quantified. Protein (30 μg) was separated in an SDS-PAGE gel and then electrotransferred onto a PVDF membrane (0.22 μm pore; Millipore, USA). Membranes were incubated with primary antibodies overnight at 4 °C with subsequent incubation with secondary antibodies labeled with horseradish peroxidase. The immunoblots were detected by an enhanced chemiluminescent substrate (Millipore) on the ChemiDoc MP system (Bio-rad, USA). Primary antibodies: BDNF (rabbit, 1/500, Abcam, Cambridge, UK); PAR-2 (mouse, 1:1000, Santa Cruz, USA), phospho-p38 (Thr180/Tyr182), p38, phospho-p65 (Ser536) and p65 antibodies are from Cell signaling Technology, USA (Rabbit, 1/1000); GAPDH (1:1000, Beyotime, Nanjing, China); horseradish peroxidase-conjugated anti-rabbit/mouse secondary antibodies (1:10000, Zhongshan Gold Bridge, Beijing, China).

### Animals

All experimental protocols for the animal study were approved by the Animal Care and Use Committee of Shandong University (Document No.201202023) and all experimental procedures were carried out in accordance with the Animal Management Rules of the Chinese Ministry of Health (Document No. 55, 2001) and guidelines of the International Association for the Study of Pain.

Male C57BL/6 mice were maintained in transparent plastic cages in a temperature-, light- and hygrometry-controlled room (20 ± 2 °C, 12: 12 hours light/dark cycle, 50 ± 5%) at the key Laboratory of Cardiovascular Remodeling and Function Research, Qilu Hospital of Shandong University.

### Intracolonic infusion, colorectal distension (CRD) and electromyography recording (EMG)

Mice were pre-implanted with silver electrode according to Julie’s methods^29^. Mice with a surgery had 5 days for recovery, and fasted for 24 h before intracolonic infusion, CRD and EMG.

Mice received intracolonic infusion of saline or FSN (0.3 ml, 170 μl/h) from HCs or IBS-D patients, or IBS-D FSN preincubated with FUT-175 through a catheter (outside diameter 1 mm) inserted into the colon 3.5 cm from the anus. To block PAR-2 and TrkB receptors of colon, we pretreated mice by intracolonically administration of ENMD-1068 (4 mg/mouse, 30 min before FSN infusion, Sigma) and TrkB/Fc (10 ng/mouse, 30 min before FSN infusion, R&D, USA) respectively (All residual solutions within colon were eliminated by gravity before FSN infusion).

CRD started at 1 hour after intracolonic infusion. EMG activity was used to evaluate the visceromotor response of mice and recorded by Powerlab BL-420E system. Data as expressed by area under the curve were analyzed by Labchart v5.0 software. Basal EMG activity was subtracted from values recorded during distension.

### Measurement of BDNF

Colonic tissues (at infusion area) were collected from each group of mice 1 h after infusion of supernatants. BDNF was detected by western blotting and immunostaining.

### Statistics

All data were presented as mean values ± standard error of the mean (SEM), using GraphPad Prism 6.0 c. Two-group differences were determined by independent Student t test, and multiple comparisons by one-way ANOVA test, followed by Tukey’s post-hoc tests. Two-tailed *P* values < 0.05 were taken as significant difference.

## Author Contributions

P.W. performed the experiments, analyzed the data and drafted the manuscript. F.X.C., C.D., C.Q.L., Y.B.Y. and X.L.Z. performed the experiments and interpreted the data. Y.Q.L. obtained the funding, designed the study and critically revised the manuscript. All authors have reviewed the manuscript and given advices.

## Additional Information

**How to cite this article**: Wang, P. *et al.* Increased production of BDNF in colonic epithelial cells induced by fecal supernatants from diarrheic IBS patients. *Sci. Rep.* **5**, 10121; doi: 10.1038/srep10121 (2015).

## Supplementary Material

Supplementary Information
