# Supplementary material for: Increased production of BDNF in colonic epithelial cells induced by fecal supernatants from diarrheic IBS patients
Source: Sci Rep. 2015 May 22;5:10121. doi: 10.1038/srep10121 (PMC4441152; doi:10.1038/srep10121)

Increased production of BDNF in colonic epithelial cells induced by fecal supernatants from diarrheic IBS patients

Peng Wang[1, 2], Fei-Xue Chen [1, 2] , Chao Du[1, 2], Chang-Qing Li[1, 2], Yan-Bo Yu[1, 2], Xiu-Li Zuo[1, 2], Yan-Qing Li*[1, 2]

[1]Department of Gastroenterology, Qilu Hospital, Shandong University, Jinan 250012, P.R. China

[2]Laboratory of Translational Gastroenterology, Qilu Hospital, Shandong University, Jinan 250012, P.R. China

***Correspondence**

Yan-Qing Li, M.D., PH.D.

Department of Gastroenterology, Qilu Hospital, Shandong University, Jinan 250012, China

E-mail: liyanqing@sdu.edu.cn

Tel: +86-531-82169508

Fax: +86-531-82169236

Supplementary Figure S1

Full scan images of the immunoblots. In several cases, selected data were shown in the manuscript and the PVDF membranes for immunoblots were cut into strips to minimize the amount of antibodies that are necessary for analysis. Scans of entire PVDF strips are provided.

Figure 3a

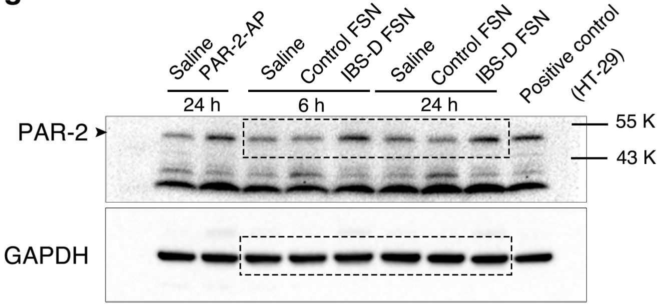

Figure 3c

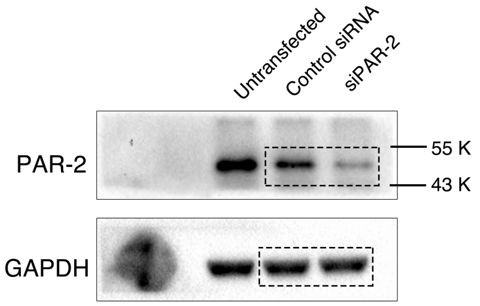

Figure 3e

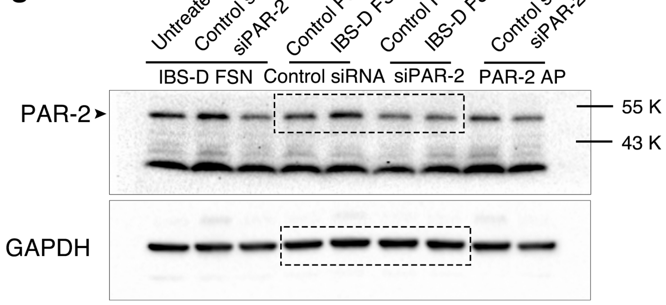

Figure 4a

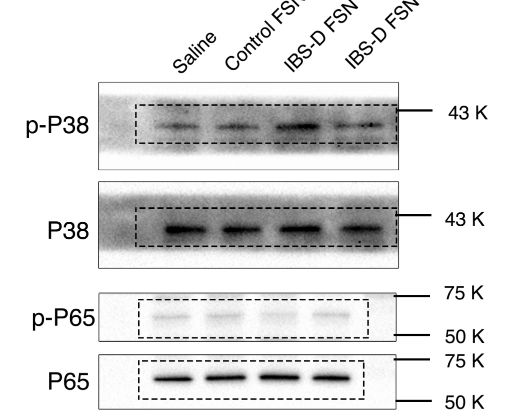

Figure 5a

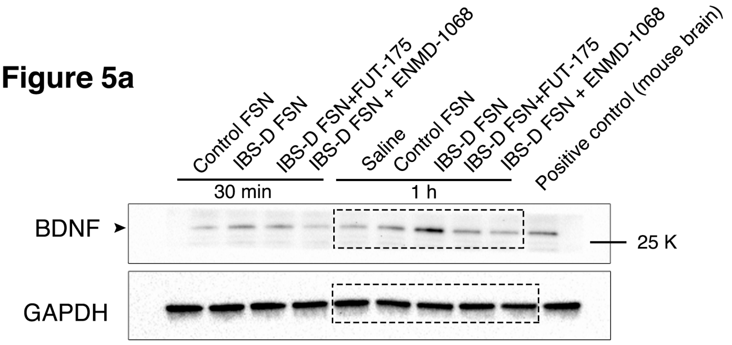

Supplement: Supplementary Information [file srep10121-s1.pdf]
